# Electrical characterization and nanoscale surface morphology of optimized Ti/Al/Ta/Au ohmic contact for AlGaN/GaN HEMT

**DOI:** 10.1186/1556-276X-7-107

**Published:** 2012-02-07

**Authors:** Cong Wang, Nam-Young Kim

**Affiliations:** 1 RFIC Centre, Kwangwoon University, 617-2, Bima-Kwan, 26 Kwangwon-Gil, Nowon-Ku, Seoul, South Korea

**Keywords:** ohmic contact, contact resistance, surface morphology, edge line definition, high electron mobility transistor

## Abstract

Good ohmic contacts with low contact resistance, smooth surface morphology, and a well-defined edge profile are essential to ensure optimal device performances for the AlGaN/GaN high electron mobility transistors [HEMTs]. A tantalum [Ta] metal layer and an SiN_x _thin film were used for the first time as an effective diffusion barrier and encapsulation layer in the standard Ti/Al/metal/Au ohmic metallization scheme in order to obtain high quality ohmic contacts with a focus on the thickness of Ta and SiN_x_. It is found that the Ta thickness is the dominant factor affecting the contact resistance, while the SiN_x _thickness affects the surface morphology significantly. An optimized Ti/Al/Ta/Au ohmic contact including a 40-nm thick Ta barrier layer and a 50-nm thick SiN_x _encapsulation layer is preferred when compared with the other conventional ohmic contact stacks as it produces a low contact resistance of around 7.27 × 10^-7 ^Ω·cm^2 ^and an ultra-low nanoscale surface morphology with a root mean square deviation of around 10 nm. Results from the proposed study play an important role in obtaining excellent ohmic contact formation in the fabrication of AlGaN/GaN HEMTs.

## Introduction

AlGaN/GaN high electron mobility transistors [HEMTs] are a promising technology for high-frequency, high-temperature, and high-power electronic devices due to the fact that AlGaN/GaN HEMTs have high breakdown voltage, high sheet carrier density, and high saturation current [1-4]. The formation of low-resistance ohmic contacts is a key issue in the fabrication process of AlGaN/GaN HEMTs, which are also required to have a smooth surface morphology and well-defined edge acuity [5-7]. The conventional ohmic contact metal uses a Ti/Al-based Ti/Al/Ti/Au or Ti/Al/Ni/Au structure [8-11]. Ti is essential because it participates in the reaction at the interface with nitrides to form TiN or AlTi_2_N layers by high-temperature rapid thermal annealing [RTA], and nitride vacancies are simultaneously formed at the AlGaN/GaN surface. At the same time, the diffused Ti and Al reduce the native gallium oxide on the AlGaN/GaN surface [12]. Au is applied as an outer layer to prevent the oxidation of the Ti/Al metals during the RTA process. In addition, a diffusion barrier layer (Ti or Ni) is applied to prevent or minimize the Au upper layer from diffusing downward. However, this scheme often exhibits a very bumpy surface morphology and a significant lateral overflow during alloying at high temperature due to the intermixing of the Au and Al, which forms a viscous AlAu_4 _phase at high annealing temperatures. In order to minimize this phenomenon, special Au diffusion barrier layers, such as Pt [13] and Mo [14], and various studies regarding such factors as Ti/Al metal thickness adjustment, RTA condition changes, ion implantation annealing, recess etching of the ohmic contact, and epi-layer optimization have been carried out [15-17]. Unfortunately, these methods cannot solve all the problems of low contact resistance, smooth surface morphology, and well-defined line edge issues simultaneously.

In this paper, we present a report on an optimized Ti/Al/Ta/Au ohmic contact metallic system with an application of SiN_x _encapsulation layer before the annealing process, which results in a significantly better ohmic contact behavior for the AlGaN/GaN HEMTs. We present the comparative electrical, morphological, and microstructural properties for different ohmic metallic stacks. It has been found that the proposed ohmic metallization scheme using Ti/Al/Ta/Au with an SiN_x _encapsulation layer can provide a reliable solution for AlGaN/GaN HEMT applications by supplying superior electrical, morphological, and microstructural properties.

## Experimental details

**Figure 1:**
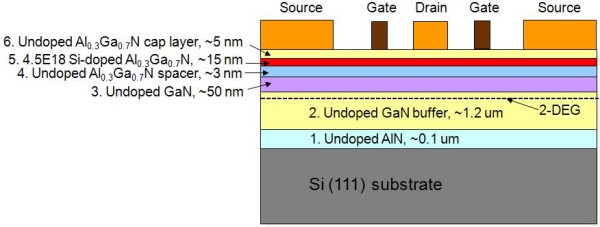
**Schematic of the AlGaN/GaN HEMT on the Si (111) substrate**.

The epitaxial layers used in this work are grown using metal organic chemical vapor deposition on an Si (111) substrate, composed of a GaN buffer layer, followed by a 50-nm undoped GaN layer, a 3-nm undoped Al_0.3_Ga_0.7_N spacer, a 4.5 × 10^18^-cm^-3 ^Si-doped Al_0.3_Ga_0.7_N layer, and capped by a 5-nm undoped Al_0.3_Ga_0.7_N layer. The mesa isolation was defined by inductively coupled plasma reactive ion etching [ICP-RIE] for 35 s using Cl_2_/BCl_3 _(6:1) mixing gas; the etching depth obtained was at least 200 nm. Various ohmic contact metallization structures were deposited by electron beam evaporation with a base pressure of 1 × 10^-8 ^Torr and patterned by the lift-off process. Before evaporation, the surfaces of the samples were treated by a HCl/H_2_O = 1:10 solution and smooth O_2_/H_2 _plasma etching at 80°C for 60 s in order to remove the native oxide layer. The specific contact resistance [*ρ*_c_] was measured through transmission line method measurements; the square (100 × 100 μm^2^) contacts were separated by 2, 4, 8, 16 and 32 μm. The surface morphology was characterized using a scanning electron microscope [SEM] and an atomic force microscope [AFM]. The root mean square [RMS] value for surface roughness of these metal contacts was measured from the analysis of a 10 × 10-μm^2 ^area on the ohmic metal pattern. The exposed components of ohmic contact were detected using energy dispersive spectrometry [EDS] spectra. The schematic diagram of the AlGaN/GaN HEMT sample is shown in Figure 1.

## Results and discussion

**Figure 2:**
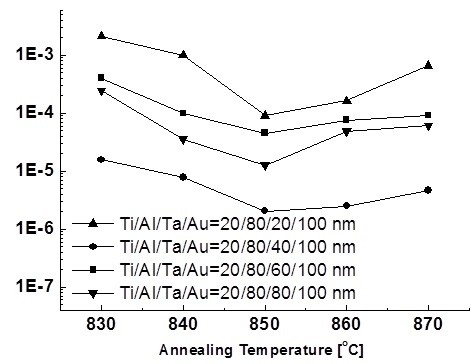
**Temperature dependence**. The temperature dependence of the specific contact resistance for Ti/Al/Ta/Au ohmic contact stacks with different Ta thicknesses in the transmission line method [TLM] test structure on various annealing temperatures.

At the initial experiment, Ti/Al/Ta/Au ohmic metal stacks with different Ta thicknesses (20, 40, 60, 80 nm) were used and annealed in an N_2_-flowing ambient at various RTA temperatures for 30 s. The thickness of Ti, Al, and Au were fixed with 20, 80, and 100 nm, respectively. Figure 2 shows the typical behavior of the *ρ*_c _values as a function of the annealing temperature for Ti/Al/Ta/Au ohmic contact systems with different Ta thicknesses. As shown in Figure 2, the *ρ*_c _decreases as the annealing temperature increases from 830°C to 850°C, and the lowest value of 2.03 × 10^-6 ^Ω·cm^2 ^is obtained when the Ta thickness is 40 nm. Therefore, Ta thickness is the dominant factor affecting the *ρ*_c _of Ti/Al/Ta/Au ohmic metal stack, and a Ta with 40-nm thickness is preferred for achieving ohmic contact with low contact resistance.

**Figure 3:**
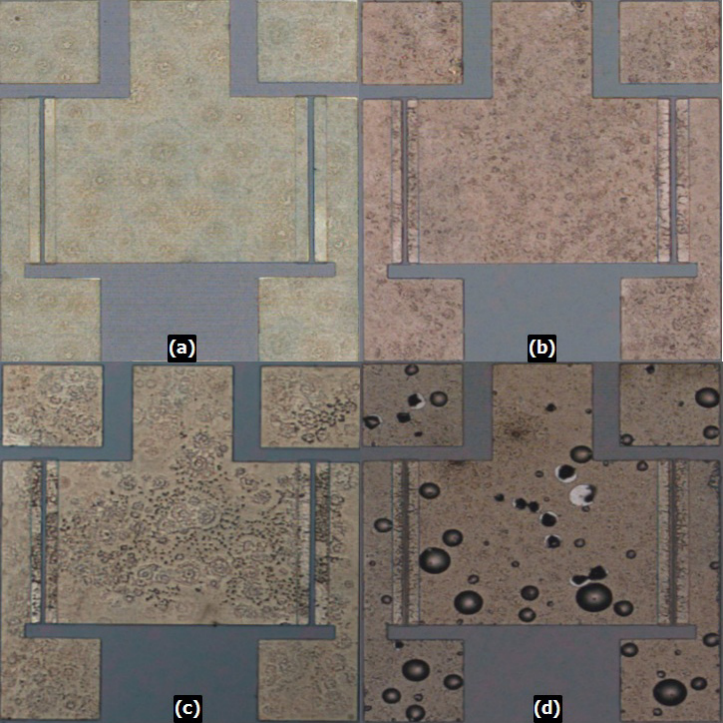
**Microscope photographs of surface morphology**. Microscope photographs of surface morphology of Ti/Al/Ta/Au (20/80/40/100 nm) ohmic stacks with different SiN_x _thicknesses after annealing at 850°C for 30 s: (**a**) with 50-nm SiN_x _encapsulation layer, (**b**) 100-nm SiN_x _encapsulation layer, (**c**) 150-nm SiN_x _encapsulation layer, and (**d**) 200-nm SiN_x _encapsulation layer.

In order to evaluate the influence of the SiN_x _encapsulation layer on the performance of the ohmic contact, a divided thickness of 50, 100, 150, and 200 nm of SiN_x _was deposited by plasma-enhanced chemical vapor deposition [PECVD] using 320/9/2,000 sccm of SiH_4 _(5%) + He (95%)/NH_3_/N_2 _mixing gas, an RF power of 100 W, a chamber pressure of 1200 mTorr, and a chamber temperature of 250°C on the Ti/Al/Ta/Au (20/80/40/100 nm) ohmic metal. Figure 3 shows a visual surface morphology of the process control monitor pattern with different thicknesses of SiN_x _after the RTA process. As shown in Figure 3, the surface morphology decreases as the SiN_x _thickness increases from 50 to 200 nm. An encapsulation layer of SiN_x _with a 50-nm thickness is an effectual way to obtain ohmic contact with smooth surface morphology.

**Figure 4:**
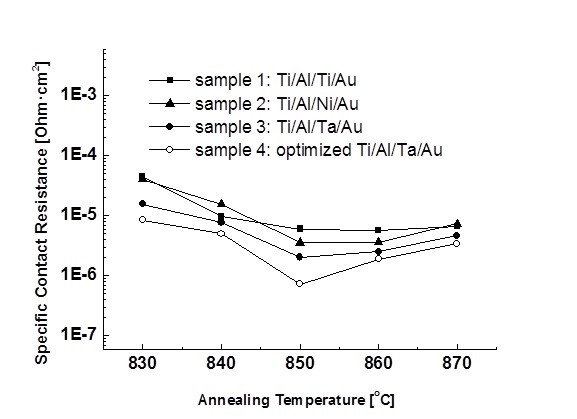
**The temperature dependence of the specific contact resistivity for all samples**. Sample 1 (filled square): Ti/Al/Ti/Au; sample 2 (filled triangle): Ti/Al/Ni/Au; sample 3 (filled circle): Ti/Al/Ta/Au; and sample 4 (empty circle): the optimized Ti/Al/Ta/Au metallization ohmic pads in TLM test structure on various annealing temperatures.

**Figure 5:**
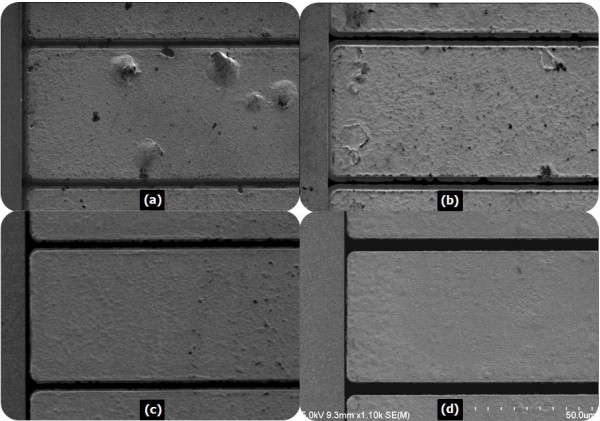
**The SEM photographs of the surface morphology**. The SEM photographs of the surface morphology with various ohmic contact systems after annealing at 850°C for 30 s: (**a**) sample 1, Ti/Al/Ti/Au; (**b**) sample 2, Ti/Al/Ni/Au; (**c**) sample 3, Ti/Al/Ta/Au; and (**d**) sample 4, the optimized Ti/Al/Ta/Au. The images show the metal on either side of a 5-μm gap.

After fixing the thickness of the barrier layer Ta in the Ti/Al/Ta/Au ohmic contact stack and the thickness of the encapsulation layer SiN_x_, four different ohmic metallization schemes having the same metal thickness (20/80/40/100 nm) were prepared for comparison: sample 1 was Ti/Al/Ti/Au, sample 2 was Ti/Al/Ni/Au, sample 3 was Ti/Al/Ta/Au, and sample 4 was the optimized Ti/Al/Ta/Au with a 50-nm SiN_x _encapsulation layer. The as-deposited samples were then introduced into an RTA system that ranged from 830°C to 870°C for 30 s under an N_2 _atmosphere. After the RTA processing, the SiN_x _encapsulation layer was etched out using a dry/wet etching method in order to avoid damage to the epitaxy materials. First, 40 nm of the layer was dry etched by the low-damaged ICP-RIE, and then the remaining 10 nm was wet etched by a 1:6 buffer oxide etch solution. It has been found that PECVD-deposited SiN_x _can be successfully removed without damaging the contact metal and the epi-layer after a high-temperature RTA process. Figure 4 shows the typical behavior of the *ρ*_c _as a function of the annealing temperature for the four ohmic contact systems. As shown in Figure 4, a better contact resistance is detected when the Ta is used as an effective diffusion barrier, and the lowest *ρ*_c _value of 7.27 × 10^-7 ^Ω·cm^2 ^is obtained in sample 4 at an annealing temperature of 850°C and an annealing time of 30 s. It is noted that after annealing, samples 3 and 4 with the Ta diffusion barrier have significantly less metal inter-diffusion when compared with samples 1 and 2. A very limited in-diffusion of Au and out-diffusion of Ti and Al occurred through the Ta barrier. All of these factors resulted in a low contact resistance. SEM was used to characterize the film smoothness and edge acuity. The Ti/Al/Ti/Au ohmic contact developed a very rough surface morphology and considerably deteriorated edge acuity. Semispherical bulges possessing various sizes were found to be distributed randomly on the surface, some of which had cracks and breaks (see Figure 5a). The Ti/Al/Ni/Au had a slightly superior surface smoothness, but the edge of the contact metal became considerably wider after annealing at 850°C for 30 s (see Figure 5b) when compared with the Ti/Al/Ti/Au contact.

We posit that the Ti-Al or Ni-Al alloy aggregation in some local areas is the reason for the poor surface formation, and these precipitated Ti-Al or Ni-Al droplets are surrounded by Au-Al alloys. The surface morphology and edge acuity were improved in the Ti/Al/Ta/Au-based ohmic metallization as can be observed in Figure 5c,d. This could be due to the fact that the high melting point of metal Ta is used as a diffusion-impervious layer to suppress the inter-diffusion and reaction between the Au and Al layers in the contact stack which are the main causes of poor surface smoothness and edge acuity.

**Figure 6:**
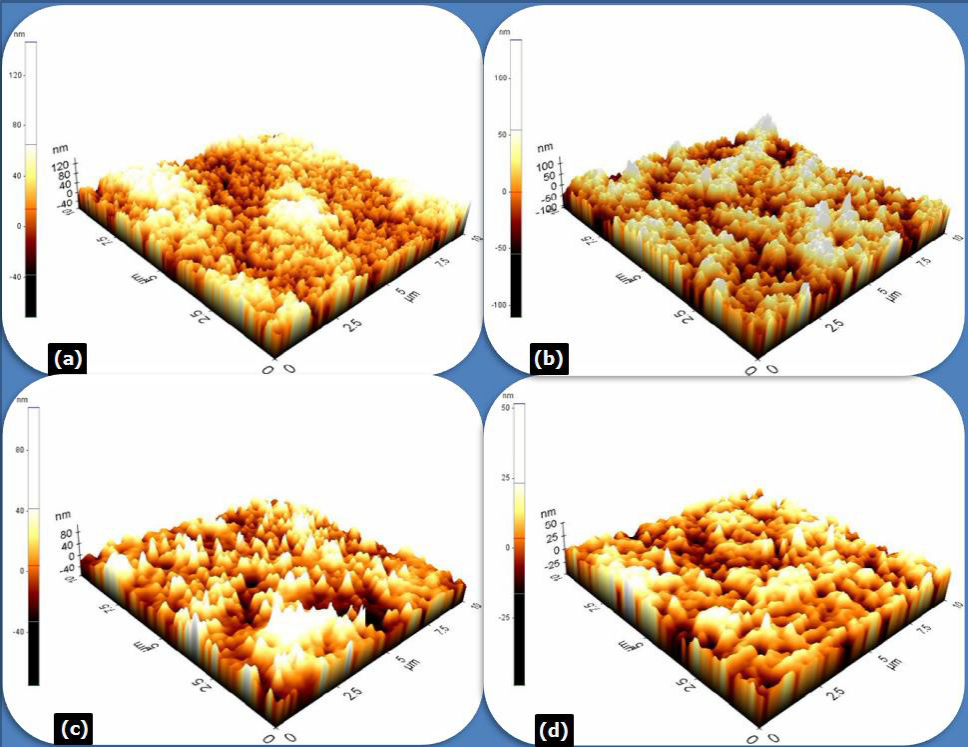
**The three-dimensional [3-D] AFM images of the surface morphology**. The 3-D AFM images of the surface morphology with various ohmic contact systems after annealing at 850°C for 30 s: (**a**) sample 1, Ti/Al/Ti/Au with an RMS value of 41.223 nm; (**b**) sample 2, Ti/Al/Ni/Au with an RMS value of 27.84 nm; (**c**) sample 3, Ti/Al/Ta/Au with an RMS value of 19.014 nm; and (**d**) sample 4, the optimized Ti/Al/Ta/Au with a RMS value of 10.006 nm. The images are 3-D AFM of 10 × 10-μm areas.

**Figure 7:**
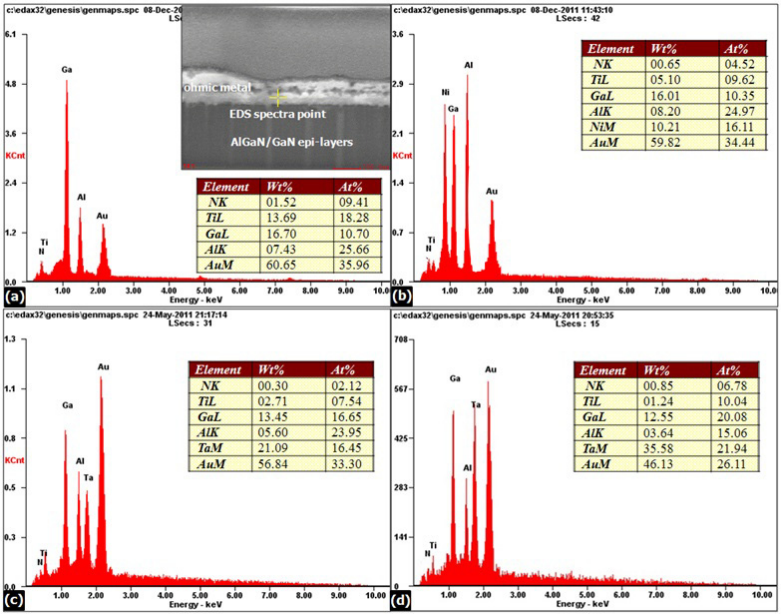
**Spectral analysis**. The EDS spectra analysis for (**a**) sample 1: Ti/Al/Ti/Au; (**b**) sample 2: Ti/Al/Ni/Au; (**c**) sample 3: Ti/Al/Ta/Au; and (**d**) sample 4: the optimized Ti/Al/Ta/Au after annealing at 850°C for 30 s (the FIB image of the cross-sectional ohmic contact with the EDS spectra measuring point is shown in the upper left of (a), and the distance from the AlGaN/GaN layers to the alloyed ohmic contact surface is 80 nm, which is fixed to all of the measured samples).

AFM was performed on the fabricated samples in order to check the surface roughness. Figure 6 shows the comparison of the surface morphology among the four sets of samples annealed at 850°C for 30 s. Consentaneous surface morphology information between the SEM and AFM can be obtained. A continuous increase in the metal roughness can be seen upon moving from sample 1 (see Figure 6a) to sample 4 (see Figure 6d). The RMS value of the surface roughness for the optimized Ti/Al/Ta/Au contact was only around 10 nm, which is significantly lower than the previously reported data of over 40 nm for Ti/Al/Ni/Au contacts [10,17], and 36 nm for Ti/Al/Mo/Au [18]. The ohmic contact performances of the Ti/Al/metal/Au schemes differ and are dependent on the nature of the metal barrier layer used. The fragmentation of the metal barrier layer into bulges has a strong implication on the nature of lateral flow. These bulges could act as physical impediments for the lateral flow of the Al-Au solid solution. The scheme with Ta is the most stable in terms of intermetallic reactions and surface morphology. Correlation between low contact resistance and low RMS value has been observed. Pretorius et al. [19] have presented a thermodynamic interpretation of the possible interactions that can take place between Al and many of the metal barrier layers. The effective concentration of the elements at the growth interface is taken to be that of the lowest temperature eutectic of the binary systems, and predictions were made about the effective heat of formation of various binaries: TiAl_3 _of -2.96 kJ/mol, NiAl_3 _of -5.32 kJ/mol, MoAl_12 _of -3.9 kJ/mol, and TaAl_3 _of -2.40 kJ/mol. The effective heat of formation was shown to be successful in correctly predicting the first and most thermodynamically feasible phases to form in metal-Al binaries. It can be clearly seen that Ta-Al alloy formation has among the lowest effective heats of formation. EDS experiments were conducted for all of the samples with the distance from AlGaN/GaN layers to the alloyed ohmic contact surface of 80 nm (Al area). Comparing the EDS analysis, a continuous decrease in the atom percentage of Al can be seen upon moving from sample 1 (see Figure 7a) to sample 4 (see Figure 7d); it is obvious that a good deal of the Al in sample 4 diffuses to the AlGaN and SiN_x _surface to create N vacancies and simultaneously reacts with the Ti and Au to confirm the formation of TiAl and AuAl phases. The concentration of N vacancies led to heavy doping and, hence, reduction in contact resistance [20]. In addition, significant intermetallic diffusion and intermixing occurred due to the fact that in-diffusion of the Au towards the Al interface is detected with the atom percentages of 35.96 (sample 1), 34.44 (sample 2), and 33.3 (sample 3) rather than that of 26.11 found in sample 4, which is a possible reason for the improvement of the surface morphology and edge line definition in sample 4. The observed experiments strongly suggest that the optimized Ti/Al/Ta/Au ohmic scheme combines the potential of low contact resistance with superior surface morphology and edge acuity.

## Conclusions

An optimized Ti/Al/Ta/Au metallization method has been successfully realized that yields improved contact characteristics that are superior to those of the conventional Ti/Al/Ti/Au or Ti/Al/Ni/Au scheme for AlGaN/GaN HEMTs. The effect of the thickness of Ta and encapsulated SiN_x _film for the proposed Ti/Al/Ta/Au contact has been studied. We have shown that the Ti/Al/Ta/Au metallization scheme encapsulated by a thin SiN_x _film before the annealing process leads to an excellent ohmic formation that not only obtains a low contact resistance, but also has a smooth morphology and favorable edge line definition. This optimized ohmic contact is most suitable in meeting the requirements of performance improvement for high-power AlGaN/GaN HEMT applications.

## Competing interests

The authors declare that they have no competing interests.

## Authors' contributions

CW conceived of the study, carried out the ohmic contact experiment, and drafted the manuscript. N-YK participated in the conception, guided the study, and revised the manuscript. All authors read and approved the final manuscript.
